# 

**Published:** 1875-04

**Authors:** 


### INDEX.

### Vol. XI to XIII inclusive

**Contributions.** PAGE.

American Phar. Ass'n—Pharmasist ... 60
Brains vs. Bowels, Ben. H. Pratt 1
Boys, How Made Bad, Ben. H. Pratt ... 281
California, S. O. Gleason, M. D... 34
Compromises with Nature, Ben. H. Pratt ... 141
Choose Ye Which, Ben. H. Pratt. 253
Dr. Ned's Reflections, Dr. Ned... 90
Doctors, I—Dr. Ned ... 115
Doctors, II—Dr. Ned ... 144
Doctor's Responsibility, Ben. H. Pratt ... 197
Foundered, P. H. Hayes, M. D... 118
Gastronomics, Ben. H. Pratt ... 86
Habit, B. Browne ... 33
Human Monster, A. R. Kilpatrick, M. D. ... 88
Hypochondriacs, Ben. H. Pratt.. 169
How to be Sick, Ben H. Pratt ... 309
Joys of Misery, Ben. H. Pratt... 58
Keeping up Appearances, Ben. H. Pratt ... 225
Man's Place in Creation, P. H. Hayes, M. D. ... 31
Metric System, M. S. Bidwell ... 174
Nerve Storms, P. H. Hayes, M. D. 172
Nerve Storms, II.—P. H. Hayes, M. D ... 199
Nurses and Neighbors. Thomas K. Beecher ... 285
Op den Graeff ... 61
Our National Diseases, P. H. Hayes, M. D ... 87
One Health, One Disease, P. H. Hayes, M. D ... 255
One Health, One Disease, II, P. H. Hayes, M. D ... 283
Only a Boy, B. Browne ... 314
Paralysis of Liver, P. H. Hayes, M. D ... 312
Probably and Perhaps, Thomas K. Beecher ... 228
Prophylaxis of Typhoid Fever, J. A. Hall, M. D ... 117
Paralysis of Stomach P. H. Hayes, M. D ... 143
Probabilities, B. Browne ... 257
Recreation Without Damnation, Ben. H. Pratt ... 30
School Life and Nerve Disease, P. H. Hayes, M. D ... 227
Salvation, Health, Thomas K. Beecher ... 4
Soda, J. B. C. ... 315
Surgery vs. Medicine, Wm. Tod Helmuth, M. D ... 316
The Currency, Farmer ... 314
The Authority, Ben. H. Pratt ... 113
The Fly, B. Browne ... 146
Waste of Life, P. H. Hayes, M. D. 3
Wages of Law, P. H. Hayes, M. D ... 59
What Do You Charge, Thomas K. Beecher ... 171

**Medical Items and News.**

Asthma ... 6–62–202–206–211–235
Anti-Constipation Pills ... 36
Anti-Spasmodic Pills ... 64

PAGE.
Antidotes ... 62–65–70–176–202–262
Arsenic ... 65–178
Arsenic Smoking ... 325
Arnica ... 65
Anto-transfusion ... 318
Abscess ... 66
Anti-Catarrhal Pills ... 93
Atropia ... 121–261
Anal Fissures ... 122
Anthelmintics ... 123
Apocynum Cannabinum ... 124
Ague ... 149
Amenorrhœa ... 176–291
Anæsthetics ... 177–181–258–261
Angina Pectoris ... 179
Aspiration ... 180–236
Anæsthesia, Bonwell's Method ... 181
Aneurism ... 204
Acne ... 234
Anæmia ... 259
Apomorphia ... 260
Anthrax Maligna ... 287
Alcohol Lesions ... 289
Antiseptic Vinegar ... 290
Bedsores ... 6
Bronchitis ... 7–10–71–96–230–289
Biliary Calculi ... 7–62–68–95
Burns ... 8–95–204–208–266
Burns and Scalds ... 40
Boils ... 37–97
Belgian Medical Laws ... 93
Blistering Substance ... 121
Bowel Affections ... 151
Belladonna Poisoning ... 176
Blood Test ... 178
Buboes ... 205
Bumstead's Mixture ... 211
Biniodidi Mixture ... 211
Blisters ... 231
Bromide of Potassium ... 232
Bladder, Aspiration of ... 236
Brain Stimulant ... 260
Bacteria ... 266–287
Bladder, Atony of ... 288
Blood, Subcutaneous Injection of 291
Cystitis ... 318
Catarrh Bladder ... 7–319
Cockroach in Dropsy ... 319
Cracked Nipple ... 320
Children, Tonic for ... 321
Chafing in Infants ... 321
" of Vulva ... 321
Constipation ... 322
Chloride of Calcium ... 323
Chloral Hydrate, Injection of ... 10–38
Coryza ... 6
Chilblains ... 6–232–261–321
Cough Mixture ... 7–230–233
Catarrh ... 11–41–67
Chorea ... 11–96–124
Constipation ... 36–123–289
Colds ... 37–98–148–178–233–261
Croton Oil Paint ... 37–289
Carbolic Acid Inhilations ... 38
Cod Liver Oil Mixture ... 39–64–96
Chloral Hydrate and Calabar ... 40
Carbonic Acid Gas ... 324
Carbolic Acid, Antidote ... 62
Carbuncle ... 62
Chloral, Administration of 62–64–149 ... 205–235–266–287–290
Camphor Ointment ... 63–233

PAGE.
Cystitis ... 63–209–261
Croton Chloral Hydrate ... 64–66
Cancer ... :67–93–120–121–204–287–293
Court Plaster ... 120
Carbolic Acid ... 120
Calabar Bean ... 123
Chloral as Surgical Dressing, 126–206
Cosmetic Lotion ... 148
Corpulence ... 178
Chloroform Narcosis ... 36–70–292
Chloroform, mode of Administering ... 180–233
Chloroform Liniment ... 204
Coca ... 202
Convulsions ... 210
Creasote ... 235
Cholera Infantum ... 260
Cod Liver Oil, substitute for ... 263
Camphor Vapor Baths ... 289
Cosmoline ... 291
Dropsy of Joints ... 6
Diphtheria, 6–6–8–18–43–94–180–235–258 ... 260–264–267–288–327
Diphtheria, origin of ... 319
Dyspepsia ... 7–64–263
Dysentery ... 8–33–69–93–318
Dentifrice ... 37
Diarrhœa ... 64–65–66–67–99–211–214–318
Diabetes ... 68–127–151–153–207–213
Delirium Tremens ... 69–148
Dentition, painful ... 121
Drunkenness ... 121–177
Dropsy ... 124–203–235–321–349
Discoloration by Oxide of Silver ... 125
Death, Indication of ... 203
Depilatory ... 233
Disinfectant ... 234
Digestive Pastilles ... 263
Dublanchet Dr ... 287
Dental Caries ... 320
Dysmenorrhœa ... 322
Drowsiness ... 318
Enlargement of Spleen ... 6
Epistaxes ... 6
Epilepsy ... 39–127–177–209–230
Eczema ... 93–122–176
Erysipelas ... 94–211–213–232
Ergot ... 37–122–213–230–258–288
Ergotine ... 122
Eucalyptus ... 149–203–235
Emmenagogue Pill ... 204
Embalming ... 207
Expectorant Mixture ... 211
Expectorants ... 260
Elixers ... 258–259–260
Ether, Dangers of ... 292
Fumigator ... 37
Fever ... 68–262
Fish Diet ... 94
Febrifuge ... 176–203
Freckles ... 182
Fetid Feet ... 231
Fractures ... 232
Feeding of Infants ... 265
Gonorrhœa ... 8–36–210–230–259
Gastro Enteritis ... 70–213
Gelseminum ... 125
Grapes in Fever ... 148
Glandular Swellings ... 210
Galactagogue ... 231
Gout ... 233–288
Guaiaci Viridis Mist ... 289

PAGE.
Germs in Disease...319
Hemorrhoids..6-36-63-236-260-288-322
Hydrocele...6-38-121-176
Hepatitis...7
Hysterical Spasms...9
Hay Fever, cause of...10
Hay Fever...324
Hæmostatic...36-202-259
Hypodermic Injection Ergot...37-230
Hemorrhage in Extracting Teeth. 39
Hemorrhage, Post-Partum...120
Headache...41-261
Hydrocephalus...63
Hysteria...69-93
Hiccough...120-148
Hoarseness...121-230-259
Hæmoptysis...128-230
Herpes Zoster...128
Hard Case...148
Hydrophobia...149-150
Hydrocyanica Mixture...211
Heart Disease...212
Hydrobromic Ether...258
Hernia...262
Homœopathy in England...292
Iron...321
Itch...7-121
Itching...289
Inverted Toe Nail...10
Iodine Caustic...36
Intestinal Catarrh...40
India Ink Marks, to Remove...62
Iodized Glycerine...63
Intermittent Fever...93-203-262
Inunction...96
Intestinal Obstruction..96-125-180-208
Ice, Medicated...151
Infants, Feeding of...265
Insomnia...291
Jaborandi...121-231-322
Laryngitis...8-236
Local Anæsthesia...36-62-120
Local Anodyne...37-176
Ligatures...65
Lafayette Mixture...211
Leucocythæmia...232
Leucorrhœa...321
Liquorice Root Syrup...232
Lock-Jaw...262
Leeches, to Make Bite...262
Muriate of Morphia...321
Mania...63
Milk of Almonds...64
Malaria...122
Malarial Chills...237
Malarial Diseases...320
Morphine...128
Meningitis...262
Myrrh...263
Mustard Paper...287
Milk Diet...291
Neuralgia of Larynx...8
Neuralgia..10-120-149-202-203-233-258 ...324
Nipples, Cracked...320
Night Sweats...128
Nitrate of Zinc...183
Necrosis...213
Nervous Debility...265
Ozæna...6-36
Obstetrical Tincture...63
Oedema...93
Otorrhœa...124-152
Offensive Breath...148
Obesity...202-291
Opium...321
Pityreasis Capitis....6-94-149-177-293
Peritonetis...7-65
Pneumonia and Bronchitis, 9-95-122 ...258
Polk's Syrup Ferrous Hypophosphite Compound...9
Prurigo...37
Poultice...38-150
Plugging Posterior Nares...65
Pruritis...93-94-178-205-289
Passage of Pins...93
Polypus...95
Paralysis, Cystic...97
Percussion...99
Palpitation of Heart...120-259
Pumpkin Seeds...123
PAGE.
Pulsatilla...176
Pulmonary Gangrene...177
Pigmentary Stains...203
Polyuria...207
Puerperal Convulsions...210
Pain, Relief of...213
Priapism...230
Prickly Heat...231-259
Phthisis Pulmonalis...325
Phthisis, Expectoration of...235
Plague, The...287
Pulse by Telegraph...287
Palpitation, Functional...321
Phosphorus Pill...318
Pleurisy...321
Podophyllin Pill...321
Quinine...128
Quinia, in Small Doses...320
" for Hypodermic...322
Rheumatism...7-9-37-39-62-66-68-93 ...123-126-128-148-150-153-176-179 ...205-234-258-263-264
Ringworm...40
Raw Meat, Method of Administering...62-263
Red Face, Cure of...182
Rhus Toxicodendron...206
Rickets...208
Syphilis...6
Syrup Hypophosphite Iron Quinia and Strych...8
Sea-Sickness...8-212-258
Stricture Urethra...9
Sycosis...10-176
Stomach, to Wash Out...11
Surgical Fever...36
Sciatica...38-289
Snake Bite...41-177
Skin Grafting...63
Sore Nipples...66-207
Strychnia Antidote...70-176-202
Scirrhus...93
Sore Throat...94-260-261
Strapping...95
Scarlatina...122-127-258
Salicylic Acid, 123-124-153-176-184-204 ...213-234-264
Skin Diseases...150
Soda-Water Syrups...152
Santonine...176-230
Strychnia, Action on Eye...177
Spleen, Enlargement of...203-205
Salicylate of Ammonium...204
Salivation...204
Syrups, Preservation of...205
Sinapisms...206
Swellings, Glandular...210
Sedative Pills...230
Salicylate of Soda...233
Sea Water...260
Sunstroke...263
Sedatives for Young...264
Spinal Irritation...265
Small-Pox...290
Small Doses, Value of...293
Salicylic Acid, Solvent for...320
Tampon, to Apply...62
Toothache...64-176-202
Typhoid Fever...67-98-151-234-290
Tape Worm...71-178-261
Tobacco...95
Tippling...97
Teeth, Carious...121
Test for Blood...178
Tuberculosis...233-236
Tumors...262
Tetanus...262
Turpentine Bath...288
Tooth Filling...288
Triplex Pill...321
Taenium Soleum...319-323
Urea, Estimation of...319
Ulcers, Old...7
Ulceration of Nose...9
Urine, Examination of...72-125-149
Uric Acid, Reagentfor...177
Uterine Catarrh...202-258
Urticaria...204-231-288
Ulcers, Syphilitic...204
Urine, Incontinence of...231
Uterine Tumors...262
Uterine Hemorrhages...290
PAGE.
Vaccine Lymph...9
Varicocele...40
Variolus Pustules...177
Venereal Lesions...183
Wine of Beef and Iron...36
Whooping Cough...42-205-208-258
Warts...63
Worms...120
Wounds...124
Warburg's Tincture...260
Yellow Fever Poison...179

**Correspondence.**

Letters Exposing Quacks and Humbugs, with Other Communications of Interest to the People,..12-44-73-100-129-130 131-132-133-155-156-157-158-159-185 ...326-215-238-268-297-298

**Clippings.**

A Darwinian Plea...5
Advice to a Dyspeptic...5
Curious Blindness...92
Clairvoyance...201
Curious Facts...286
Disease, Dogs and Books as Vehicles...19
Dipsomania...19
Doctor's Carriages...54
Doctor Making...147
Flowering and Fruit Bearing Plants in Sleeping Apartments.147
Friend Indeed...305
Ice, to Keep...19
Long Canal...175
Mexican Bleeding...35
Profitable Industry...119
Quick Tanning...19
Sleeplessness...5
Scarlet Fever by Mail...19
Soft Hands...147
Smart Man...175
Sugar, granulated...317
Small Fruit in Gardens...317
Test-Paper, a New...201
Twins, What Savages Think of Them...229
Vengeance on Hamlin...92
Wife vs. Horse...305

**Household Hints & Recipes**

Apples, to Preserve...187
Apples, to Imitate Ginger...17
Apples, to Cook...15
Antidotes for Strychnia...17
Ammonia Fumes, Effect on Flowers...102
Adhesive Paper...103
Alcohol and Cold...270
Bruises in Furniture...15
Brass, to Clean...16
Butter, to Detect Fat or Lard in...47
Basket Plants...48
Bouquets, to Preserve...101
Blue, Washing...103-217
Blackboards, Coating for...161-241
Babies, Feeding with Starch...163
Bed Bugs, to Exterminate...341
Bricks, Red Wash for...241
Butter and its Substitutes...270
Blacking, French...299
Beef, to Cure...300
Chilblains...14
Chapped Hands...15-103
Cement for Metals and Glass...16
Cement for Labels...17
Cement for Brass and Wood...46
Cement for Leather...47
Cement for India-Rubber...48
Cement, for Iron...101
Cement, for Glass or China..160-270
Cement, for Gas or Steam Pipes 160
Cement, for Marble and Alabaster 161
Cement, for Vessels...216
Cement, Hot-Water-Proof...240
Cement, for Tin to Metals...240
Cider, Bottling...17
Cologne Water...46
Cosmetic Powders...75

PAGE.
Coffee....102
Castings, to Coat with Copper....104
Curry Powder....162
Copying Ink....162
Cisterns, how to build....186
Cherries, to Bottle....188
Color, to Restore....216
Copying Pencils....216
Cracks in Stoves....216
Court Plaster....217
Cooking Utensils, Poisonous....217
Carpets, Milk on....241
Corns....243-299
Chromos, to Clean....243
Camphor-Ice....269
Copal, Solvent for....269
Cocoanut Soup....269
Celery, to Stew....270
Carbon Bisulphide Lamp....299
Chairs, to Clean....300
Conservatory on House Top....300
Chloride of Lime....301
Cucumber Pickles....302
Cabbage Pickled....302
Chocolate Caramels....302
Candy, Molasses....302
Damp Walls, to Prevent....192
Diamond Cement....160
Drawings, to Fix....186
Dishes, to Wash....244
Dried Beef....270
Dried Fruit....272
Disinfectant....301-302
Experiment, Amusing....46
Egg Test....270
Eggs, Condensed....160
Eggs, to Keep....48-271
Electroplating Pewter Surfaces...101
Extract New Mown Hay....186
Engravings, to Clean....186
Eider-Down, Substitute for....244
Files, Soldering Broken....17
Feet, Sweating....46-216-299
Fence Posts, to Preserve....101
Flowers, to Freshen....104
Freckles, Lotion for....160-269
Frost-Bite....160
Fruit Eating....162
Food, Preservation of....271
Food, Infants....216
Faded Ink, to Restore....240
Fire....242
Fruit, to Dry....272
French Blacking....299
Frosting for Sprays....299
Glue, Water-Proof....162
Glue to Resist Fire....14
Ground Glass, to Imitate....46
Gas from Petroleum....161
Goulard's Lotion....161
Gilding....162-299
Globes, to Clean....240
Grease, to Remove from Marble...240
Grease, to Remove....75-160-163-244
Glass, Silvering....161
Glass Breaking, to Prevent....270
Glass, Cement for....160-270
Gilt, to Restore....299
Guarana Chocolate....301
Harness Polish....103
Hair Restorer....103
Handles Splitting, to Prevent....103
Honey, to Improve....104
Hippophagy....216
Hair and Scalp Wash....269
Hams, to Cure....300
Hair Dye, Brown....269
Hair Dye, Blonde....269
House Insects, to Extirpate....147
Iron and Wood, to Fasten....216
Iron Wire, to Give a Silvery Look. 15
Iron, to Coat with Brass....16
Ivory, to Restore....14
Ivory, to color....240
Ivory, to Render Ductile....160
Ivory, to Dye Blue....101
Ink, Red Marking....16
Ink, Black Stencil....103
Ink, Bag Marking....103
Ink, Copying....162
Ink Stains....216
Ink, Purple, Marking....216

PAGE.
Ink Marking....240
Ink, Faded....240
Ink for Rubber Stamps....269
Ink Marks, to Remove....270
Ink, Printing, to Remove....299
Ink, to Fix....299
Infant's Food....216
Indigestible Substances....271
Jelly, Chicken....102
Jelly, Arrowroot....102
Jelly, Arrowroot Wine....102
Jelly, Iceland Moss....102
Jelly Water....160
Kerosine Vessels, to clean....326
Kalsomine....186
Kid Gloves, Stained....270
Linens, to Wash....46
Labels, Adhesive....75
Lamps, to Extinguish....102
Lemonade, Flax-Seed....102
Laundry, Hint for....160
Love of Liquor, Cure for....162
Lanterns, Parisian Police....95
Leather, Vegetable....186
Lip Salve....269
Leaves, to Bleach....269
Meerschaum, Vinnese....14
Moth Preventive....15
Match Under the Microscope....47
Meat, to Tell Good from Bad....48
Marmalade, Rhubarb....75
Marking Tools....101
Mead....150
Mucilage for Labels....216
Mucilage, Pocket....101
Mucilage for Minerals....150
Milk, Kept by Chloroform....101
Milk....240
Milk on Carpets....241
Marble, Grease Spots on....240
Mirrors, to Clean....244
Mildew....244
Mildew, to Remove....104
Meat, to Keep Fresh....299
Marble, Stains on....299
Magic Lantern Slides....301
Molasses Candy....302
Nail Biting, Prevention of....136
Nickle-Plating....47-94
Oil, Illuminating....75
Oil-Cloths, to Furbish....163-270
Oil Paintings, to clean....300
Plastered Walls, to Repair....14
Pomade....14
Pudding, German Apple....15
Paste....48
Paste, for Tin....46-160
Pears, to Test Quality of....47
Potatoes, Santee....75
Potatoes, Soufflees....75
Potatoes, Paree of....75
Potatoes, a-la-Dutchesse....15
Powders, Cosmetic....75
Powders, Violet....75
Paper, Adhesive....103
Preserves, Vegetable Marrow....103
Paint, to Clean....244-269-270
Paint for Canvas....161
Paint, Cheap....187
Painting Old Buildings....162
Preserves....187
Pineapples, to preserve....187
Pears, to bottle....187
Plums, to bottle....188
Plaster, adhesive....216
Pencils, Copying....216
Powder, Tooth....217
Plaster, English Court....217
Poisonous Chewing Gum....14
Poisonous Cooking Utensils....217
Plastic Material....240
Potter's Clay for Coal Stoves....241
Purifying Sick Chamber....271
Pork, to Cure....300
Pictures, to Clean....300
Paper Hangings, to Clean....300
Pickles, Cucumber....302
" Cauliflower....302
" Cabbage....302
" Green....302
Rat Trap....17
Roses, Milk of....104

PAGE.
Red Wash for Bricks....241
Rice, to Cook....242
Rust on Steel....269
Ribbons, to Clean....269
Rubber Stamps, Ink for....269
Reseat fore the Sistum....270
Rubber, Solvent for....272
Slating, Liquid....14-241
Stings, Remedies for....16
Sponges, to Bleach....46
Starch Enamel....46
Salad, Cabbage and Ham....46
Singular Mathematical Facts....47
Soap, Erasive....75
Sick, Delicacies for....102
Stains, to Remove from Knives...160
Stains for Wood....16
Stains, Ink....216
Stains in Clothing, to Remove, 75-188
Stains, Fruit and Wine....243
Silvering Glass....161
Silver Stains, to remove....326
Scouring Liquid....163
Shoes, to make Water-tight....326
Shoe Polish, Liquid....240
Silk, Spots in....243-269
Soapstone Hearths....243
Stearine Spots....243
Spots, Paint and Varnish....243
Salve, French Lip....269
Shampoo Mixture....269
Soup, Cocoanut....269
Sweetbreads, Mock....270
Sick Chamber, to Purify....271
Straw Matting, to Clean....299
Small Pox....300
Shaving Soap....301
Tortoise Shell, Imitation of....14
Tins, to Polish....161
Tins, to Wash....243
Tin, to Remove from Copper....299
Tooth Powder....217
Tar-water Dye....270
Ventilation, Simple Plan....15
Varnish, Colorless....48
Varnish, Good....326
Varnish, Elastic Dammar....217
Violet Powder....75
Vegetable Growths on Walls....301
Waterproof Cloth....14
Waterproofing Goods....186
Wood, Red Dye for....75
Wood and Iron, to Fasten....216
Woodwork, to Clean....14
Writer's Cramp....16
Warts....46-269
Wines, to Discover if Colored....47
Washing Blue....103-217
Waterproof Fabrics....104
Wax Spots, to Remove....160
White Spar, to Clean....269
Whitewash....161-272
Whitewash for Out-Doors....272
Wine, Treatment of....242
Walks, Cheap....243
Wash for Scalp....269
Washing Suggestions....271

**Editorial.**

Angling....49
Accidents to the Eye....52
Ankle Joint....80
A German's Experience with Quacks....134
Anchylosis....164
Asthenopia....246
Alcoholic....274
Criminal Negligence....20
Cataract....76
Cholera Infantum....276
Children's Eyes....303
Clothing....305
Deformities of the Feet....21
Deformities of the Toes....22
Doctor-Making....105
Dangerous Interference with the Ears....189
Diseases of Retina and Nerve....218
Diphtheria....327
Ectropium....20-303
Ethics....188
Flour Making....135

PAGE.
Felons....................220
False and Stiff Joints....................273
Funerals....................275
Glaucoma....................105
Hard to Please....................248
Hemiopia....................276
Infants' Eyes....................133
Look Out....................23
Our Inheritance....................304
Pleasure and Iron....................108
Paralysis of Children....................245
Pets....................248
Report of Fifty Operations for Cataract....................80
Spring Fever....................24
Stomachs....................107
Spectacles....................164
Take Your Vacation....................24
The Laryngoscope....................53
Trichina Spiralis....................190
Trials of a Dyspeptic....................247

**Editorial Chit-Chat.**

A Reason for It....................25
A Success....................27
After a Ball....................55
Asked for a Letter....................82
*Advertiser's* City Editor....................82
A Puppy....................84
Æsthetic....................249
Art Parlor....................249
An Artist....................306
Autumn Leaves....................306
Behind Time....................25
BISTOURY Wanted....................81
Busy and Dirty....................137
Builders' Estimates....................168
Balancing Effort....................222
Bile Time....................249
Bill of Fare....................251
Blue Glass Mania....................251
Book, Mr. Towner's....................278
Browne, B....................306
Brigham Young....................306
Cabinet of Brains....................329
Cork in California....................25
Co-Education of the Sexes....................25
Chloroform Death....................26
Curiosity....................26
Clara Morris and the Moxa....................82
Copenhagen....................110
Compliments of the Season....................111
Christmas....................222–111
Cosmoline....................138
Centennial Times....................139
Camping Out....................166–192
Compromises with Nature....................166
California Jack....................193
Character....................193
Charity Patients....................221
Conservatory Labors....................252
Contribution, A....................307
Dissecting Room Scene....................25
Difficult Order....................25
Drunken Babies....................25
Difference in Medical Views....................26
Disagreeable....................26
Double Headed Trout....................55
Dr. Gleason's Return....................56
Doane, Dr. W. C....................109
Driving Park....................167
Doctor's Conservatory....................224
Dramatic Talent....................278
Ex-Mayor Caldwell....................25
Exercise....................56
Entertaining....................81
Elmira Driving Park....................82
Encyclopedias....................109
Elmira Water Cure....................138
Eldridge, Dr....................222
Fifth Ward Steamer....................55
PAGE.
Father Stack....................55
Flood the Low Ground....................56
Fifty-five Cents....................109
Feelingly....................111
Floral Decorations....................137
Flowers....................167
Flower Time....................249
Florists....................306
Florists....................329
Florida....................138
Florida Letter....................166
Floridians....................166
Five-Year-Olds....................166
Faithful Officials....................167
Foundered....................192
Fine Singing....................221
Floriculture....................221
Fond of It....................250
Fritz's Sayings....................329–251
Forgot It....................277
Flea, A....................308
Grateful....................27
Gems of the Dance....................249
Garden Statues....................306
Ghost, A....................306
H....................249
Holloway's Asylum....................55
Hooked Adder in Stomach....................112
Hartz....................136
How to Acquire Health....................137
H, at Last....................166
Hail to the King....................192
Huxley....................192
Horrible....................193
Hideous Chromo....................221
Horseback Riding....................249
Hay Fever....................277
Humbug, Pleasanton's....................306
Home....................307
Horse Racing....................308
Index....................329
Infinitesimal Dosing....................25
Is it You?....................136
In Arrears....................136
It Makes a Difference....................168
James Vick....................109
Johnson....................221
Jim....................307–278–330
July Number....................25
Livingston's Humerus....................25
Lecture Course....................82
Left Over....................166
Lost Votes....................192
Little Late....................249
Laborers, City....................279
Loaned Newspapers....................329
Manifestations....................26
Medical Advertising....................28
Mean Patient....................55
Mailing Machine....................81
Minature Mine....................138
Model City....................193
Micro-Photographs....................221
New Year....................329
Musical Instrument....................330
New Local....................81
New Cover....................249
New York P. M....................308
Nervous Patient....................308
Our Improvements....................27
Our Correspondence....................136
Operations, Cataract....................279
Our Delay....................279
Our Advertisers....................306
One Handed Women....................329
Pills....................25
Photograph Their Hands....................25
Patent Tent....................27
Photography....................81–277
Photographing Extraordinary....................166
Park Church....................81
Park Church Fair....................109
PAGE.
Poor Robin....................136
Pigeon Shooting....................139
Pocket Gymnasium....................140
Poem, Mr. Towner's....................166–192
Pointer....................329
Plumbers' Help....................168
Pay Up....................221
Personal....................221
Presidential Muddle....................222
Perplexed Countryman....................223
Pansies....................277
Pay....................306
Popular Science Monthly....................307
Quack Almanacs....................110
Queer Tree....................192
Quick Work....................277
Religious Insanity....................25
Reading in Bed....................83
Russian Overcoat....................109
Reminder....................109
Returned....................136
Reliable Medicines....................137
Ride on a Locomotive....................137
Receipts....................140
Railway Crossings....................277
School Hygiene....................27
Strong Inquiry....................56
State Fair....................81
Sixteen in One Bed....................82
Sectarian Schools....................83
Search in the Dark....................84
Strong Letter....................109
Surgical Institute....................110
Statue, American Girl....................167
Sunday Opening....................168
Schooner....................193
Spiritual Manifestations....................193
Spectacle Venders....................194
Street Cars....................221
Sore Finger....................222
Spring Styles....................249
Strong....................250
Sweet Forget-Me-Not....................306
Street Frights....................307
Spitting....................307
Stealing a Camel....................330
Second Marriages....................329
Spiral Shirt Studs....................330
That Winter....................26
There's Millions in It....................28
That Vacation....................55
The Fourth....................55
The Colored Company....................81
That Story....................109
Tricks of the Trade....................137
Trouting....................137
That Royal Train....................138
Trichina....................166
The Centennial....................167–194
Thrush....................167
Theodore Tilton....................192
The Difference....................221
Time....................221
The Zoo....................222
Time Absorbent....................249
Telephone....................250–306
Temperance Movement....................252
The Crops....................277
Tramps....................278
Turks, The....................307
Vehicle for Medicine....................55
Vick....................82
Vine Stub....................82
Valuable Cross....................167
Vacancy....................221
W. F. C....................81
Wrought Iron Furnaces....................83
Where, O, Where!....................136
Wise Doctors....................137
Warner, W. R. & Co....................221
Weathery....................250
Wild Flowers....................277

### NEW BOOKS, PERIODICALS, &c.

PETERSON, the most popular ladies' magazine in America, and the *cheapest*, too, only $2.00 a year, with postage paid. Chas. J. Peterson, publisher, 306 Chestnut St., Philadelphia.

WILSON'S HERALD OF HEALTH, a new Southern health journal, published monthly by Southern Publishing Co., Atlanta, Ga., at $2 per annum. Dr. Steinback Wilson is its able editor.

CHURCH'S MUSICAL VISITOR, a journal devoted to Music and the Fine Arts, reaches us occasionally. It is only $1.50 per annum, contains much valuable music, and is published monthly by John Church & Co., 66 West 4th Street, Cincinnati, Ohio.

ARCHIVES OF DERMATOLOGY, a Quarterly Journal of Skin and Venereal Diseases, edited by L. Duncan Bulkley, A. M., M. D. G. P. Putnam's Sons, Publishers, 308, 4th Avenue, N. Y. $3 per year.

FOREST AND STREAM.—Do you read it? If not, procure it, whether you are given to field sports or not. It is a high toned, gentleman's journal, and treats of out door pleasures. By cultivating such tastes among its readers, it does much good as a health journal. We read it, enjoy it, and recommend it. Address Forest and Stream Publishing Co., 17 Chatham Street, New York. Subscription price, $5 per year.

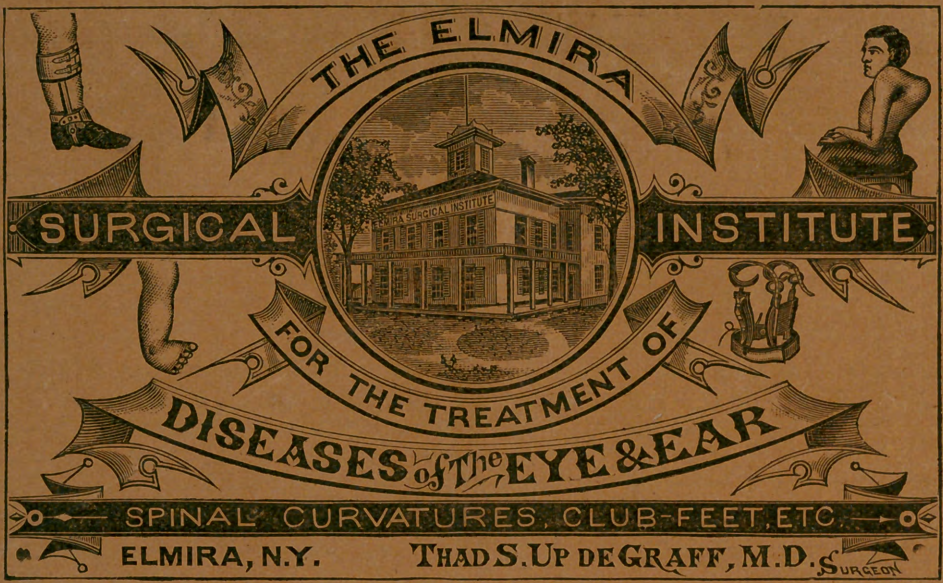

### SUBSCRIPTIONS RECEIVED

To April 1, 1875.

In this column we will credit every subscription received during the last quarter. Subscribers will please notify us immediately if their payments are not duly credited. The State only is given—this, with the full name of the party subscribing, will be sufficient to indicate who is meant:

NEW YORK.—John R Cooper, Dr W R Wells, P Coburn, Dr H A Bolles, Mrs H S Halsted, A Howard, F M Jones, H S Gilbert, Dr Wm Coryell, Jno Callister, D L Holden, Dr A E Metcalfe Jno D Hopkins, Dr M D Spencer, H W Norman, Dr H D Seaman, S H Ainsworth A Osgood, Dr F B VanAlstyne, Dr Jas H Horton, Mrs E J Smith, Dr E G Derby, U P Crane, A F Adams, Ira Brownson, E B Youmans, C J Thro, Eli Wheeler, A Houghton, Jr., N M Carter, Dr J M Hagey, W H Hawly, L D Kenfield, C M Greatsinger, Dr Wm Johnston, Walter Booth, C E Hollenbeck, Dr O C Clark, J W Henocksburg, Wm Brown, A B Abbott, W W Whitsey, Dr Sam'l S Wilcox, G D Gridley, Dr W L Appley, Dr R D Wilcox, C V Bresh A Otis, Mrs Amos Fuller, H R Rogers, Wesley Follett, Jos Armstrong, Henry Foster, Jesse Owen, S M Fassett, F G Hall, N P Fassett, S H Heldredth, Dr Ponce De Leon, W O Osborne, R N Cooley, Rev Geo Brown, Geo B Parker, Rev J M Austin, Dr Geo W Little, Wm Irish, Dr S N Badger, P G Smith, Reuben Ball, Bundy Bros, W E Hart, D C Robinson, W H Wilson, Mrs E D Snyder, H B Berry, Miss Alice M Spaulding, Dr Wm Gulick, Wm Boddy, M Brown, M S Palmer, Mrs A L Heseltine. Chas Palmer, Wm Doderer, T Foster, A B Chamberlain, J L McDowell, H B Gilbert, G B Graves, Benj Simmons, F J Amsden, Polly Caldwell, J W Taggart, S M Kinny, G D Henderson.

PENNSYLVANIA.—Dr Jno Wilson, Wm Bunyan, R J Wisner, Dr S Dickinson, J D O'Donell, Chester Williams, A Elliott, Alex Beede, S S Hicks, P Hillbish, Noah Leonard, Dr S T Davis, F J Truxell, C S Sellard, Albert G Hatter, James Miller, W C Richardson, J H Wehner, Wm Stewart, R Thomas, A Harshberger & Son, Dr M L Davis, C F Schaffle, Jas B Chandler, J R Cressinger, S S Packard, Dr Jno Romig, Thos Wright, L C Longwell, C C Finch, Dr J W Meade, Henry C Eger, H Shaw, Wm H Nicholas, V M Bovier, Dr Wm T Hall, J C Grier, Dr R S Wallace, Dr J Metz, W C Hyde, N F Glace, A J Miller, W D Gamage, W F Eckbech, Jno Hopper, Dr G B Curtis, G W Howle, Rob Snodpass, C F Maize, A A Root, Mrs J W Chapman, Dr J F Kocher. Dr G W James, Dr J C Shaffer, Geo D Hudson. Dr A S Cumming. D W Smith, D C Scovelle, Rev Reinhart Smith, Dr S W Dayton, C S Knight,

ALABAMA.—Dr J R Holley, E F Dyer, Dr J C McNeill, J O Zigler, Dr M Warren, J A Chapman,

CALIFORNIA.—Wm S Burns, Mrs A Higgins, Dr J M Logan, G L Simmons, Dr J F Gibbon.

CONNECTICUT.— J H Charlton, G H Mina.

D. C.—Edward Shaw, Surgeon General's Office, A Zoller

FLORIDA.—A J Wakefield, Dr Sol Newson, Cohem Oak, Dr E T Sabal, Geo A Panney.

GEORGIA,—Dr J H Bryan, Dr C S Strother.

INDIANA.—J S Shirley, Dr Anthorn, Dr T C Kimball Thos H Johnson, Mrs E M L Shaw, Jno Dury, J Vandergrift.

IOWA.—T UpdeGraff, J H Hazelton, L E Kelley, Dr J W Butts.

ILLINOIS.—Dr W Bradley, Dr W H Crane, Thos L Casey, Dr J A Hubbard.

INDIAN TER.—Dr O G Given.

KENTUCKY.—T L Newberry.

KANSAS.—G D Maxon.

LOUISANA.—Miss L S Hatch.

MICHIGAN.—A Garwood, Dr L R Dibble, V A Baker. Dr, C E Rulison, Dr Harvey Loomis. Dr F W Farquelle.

MINNESOTA.—J W Foss, R P Wells.

MAINE.—Geo E Brickett, E P Snow.

MARYLAND.—Jno E Snow, Dr R Myers.

MONTANA TER.—J M D Greene, W H Adsherd.

NEW JERSEY,—Mrs S P Leggett, Rev Bark, J M Atwater, H B Crosby, James F Norwood.

NEW HAMPSHIRE.—S M Emery. Mrs E S Harris, Miss Kate F Green.

NEVADA.—Jerry Whited.

NORTH CAROLINA,—Dr W B Ricks, Dr R A Gills.

NEBRASKA.—J B Rathbun.

OHIO.—D R Atwater, N B Tyler, S R Hamer, T Bradley, Jno Seuseman, Dr G W Pringle, Dr W M Cherrey.

OREGON.—Dr T N Snow.

SOUTH CAROLINA,—Jno W Ogilare.

TEXAS.—Jno Hoffman, S N Caldwell, Dr W S Robinson, F L Dickerson, E Heppenstall, B F Holt, W D Hoskins, G C Dial.

TENNESSEE.—J M Rutlege Dr Theo M Gault, Mrs Fannie Pond.

VERMONT.—Dr L D Ross.

VIRGINIA.—R Bell, Jr.

WISCONSIN.—G W Sheardown, Dr James Johnson, Dr T A Canfield.

NO NAME.—Horseheads, N. Y., Colchester, Conn.

